# Crystal structure of 1,3-di-*tert*-butyl-2-chloro-1,3,2-di­aza­phospho­rinane − a saturated six-membered phospho­rus nitro­gen heterocycle with a partially flattened chair conformation and a long P^III^—Cl bond

**DOI:** 10.1107/S2056989019004195

**Published:** 2019-04-02

**Authors:** Erik Mecke, Walter Frank

**Affiliations:** a Institut für Anorganische Chemie und Strukturchemie, Lehrstuhl II: Material- und Strukturforschung, Heinrich-Heine-Universität Düsseldorf, Universitätsstrasse 1, D-40225 Düsseldorf, Germany

**Keywords:** crystal structure, phospho­rus nitro­gen compound, six-membered heterocycle, *N*-heterocyclic phospho­rus compound, di­aza­phospho­rinane, chloro­phosphane, conformation

## Abstract

Colourless blocks of 1,3-di-*tert*-butyl-2-chloro-1,3,2-di­aza­phospho­rinane, C_11_H_24_ClN_2_P (**1**), were obtained by sublimation *in vacuo* slightly above room temperature. The asymmetric unit of the monoclinic crystal structure of the six-membered *N*-heterocyclic compound is defined by one mol­ecule in a general position. The six-membered ring of the mol­ecule adopts a cyclo­hexane-like chair conformation; the chair at one side is to some extent flattened as a result of the approximately trigonal–planar coordination of both nitro­gen atoms. In detail, this modified chair conformation is characterized by an angle of 53.07 (15)° between the plane defined by the three carbon atoms and the best plane of the two nitro­gen atoms and the two carbon atoms bound to them, and an angle of 27.96 (7)° between the latter plane and the plane defined by the nitro­gen and phospho­rus atoms. The *tert*-butyl groups are oriented equatorially and the chloro substituent is oriented axially. The P—Cl bond length of 2.2869 (6) Å is substanti­ally longer than the P—Cl single-bond length in PCl_3_ [2.034 Å; Galy & Enjalbert (1982 ▸). *J. Solid State Chem.* **44**, 1–23]. Inspection of the inter­molecular distances gives no evidence for inter­actions stronger than van der Waals forces. The closest contact is between the Cl atom and a methyl­ene group of a neighbouring mol­ecule with a Cl⋯C distance of 3.7134 (18) Å, excluding a significant influence on the P—Cl bonding.

## Abstract 2

Sublimation *in vacuo* slightly above room temperature gave crystals of the *P*-chloro-functionalized saturated six-membered *N*-heterocyclic title compound 1,3-di-*tert*-butyl-2-chloro-1,3,2-di­aza­phospho­rinane. In the crystal, no inter­actions stronger than van der Waals forces are found between the mol­ecules that neither suffer from chair conformation disorder nor from rotational disorder of the *tert*-butyl groups. Characteristic structural features are the partial flattening of the ‘cyclo­hexane-chair’ conformation at the heteroatom side of the six-membered ring and the length of the weakened P—Cl bond [2.2869 (6) Å].

## Chemical context   

Over the past two decades, *P*-chloro­functionalized *N*-heterocyclic phosphanes (NHPCls) received considerable attention, mainly as precursors of *N*-heterocyclic phosphenium ions (NHPs) that are valence isoelectronic compounds of the well-known *N*-heterocyclic carbenes (NHCs) (Papke *et al.*, 2017 ▸), but also as educts of tetra­kis­(amino)­diphosphanes (*e.g*. Bezombes *et al.*, 2004 ▸; Blum *et al.*, 2016 ▸; Edge *et al.*, 2009 ▸; Frank *et al.*, 1996 ▸), some of which reversibly dissociate to stable phosphinyl radicals (‘jack-in-the-box dipnictines’; Hinchley *et al.*, 2001 ▸), and as starting materials in the synthesis of mixed-valent tetra­kis­(amino)­tetra­phosphetes (Breuers *et al.*, 2015 ▸; Frank *et al.*, 1996 ▸). Furthermore, NHPCls and NHPs have been used as ligands in transition metal complexes (Thomas *et al.*, 2018 ▸), some of which have a potential application in catalysis (Gatien *et al.*, 2018 ▸). In the context of NHP chemistry, the majority of compounds are five-membered cycles, and especially *P*-chloro­functionalized 1,3,2-di­aza­phospho­lenes (Denk *et al.*, 1996 ▸; Carmalt & Lomeli, 1997 ▸) have gained a widespread use as precursors for 1,3,2-di­aza­phospho­lenium cations (the most prominent class of NHPs) that are weak σ-donors and strong π-acceptors (Caputo *et al.*, 2008 ▸; Tuononen *et al.*, 2007 ▸). A limited number of structurally characterized examples is known for the class of *P*-chloro­functionalized four-membered NHPCls Cl—P<(N*R*)_2_>*E* and the related NHPs. The fourth ring member >*E*, joining the class-defining Cl—P<(N*R*)_2_ fragment, is an >Si*R*_2_ group in most cases (*e.g.* Breuers & Frank, 2016 ▸; Gün *et al.*, 2017 ▸; Mo *et al.*, 2018 ▸; Mo & Frank, 2019 ▸; Veith *et al.*, 1988 ▸) but some compounds containing >C=N—*R* (Brazeau *et al.*, 2012 ▸), >B—Ph (Konu *et al.*, 2008 ▸) and >As—Cl (Hinz *et al.*, 2015 ▸) have also been synthesized and structurally characterized. In contrast to the aforementioned compounds with four- and five-membered rings, six-membered NHPs and NHPCls are less present in recent publications, although 2-chloro-1,3,2-di­aza­phophorinanes H_2_C<(CH_2_N*R*)_2_>P–Cl, for instance, have been known since the early 1970s (Maryanoff & Hutchins, 1972 ▸; Nifant’ev *et al.*, 1977 ▸). Temperature-dependent dynamical NMR investigations showed that in solution these substances are not subject to a fast conformation change, like the ring-inversion process of cyclo­hexane, and that in the predominant conformation the chloro substituent is expected to be in the axial position and the residues on the nitro­gen atoms are oriented ‘diequatorial’. This gives rise to a quite complex ^1^H-NMR spectrum with an *AA*′*KK*′*QTX* pattern (*X* = P, *AA*′*KK*′ = C_4_ and C_6_ protons, *Q* and *T* = C_5_ protons; Hutchins *et al.*, 1972 ▸). Furthermore, the number and position of the signals in the ^1^H-NMR spectrum are dependent on concentration, which was attributed to inter­molecular chlorine-exchange mechanisms. Even though this parent class of six-membered NHPCls has been known for quite some time, no crystal structure analysis has thus far been reported. Herein, we present the crystal structure of the title compound that allows for a structural comparison with the most closely related four- or five-membered NHPCls known, on one hand, and with phospha- and 1,3,2-dioxaphospha­cyclo­hexane deriv­atives, on the other hand.
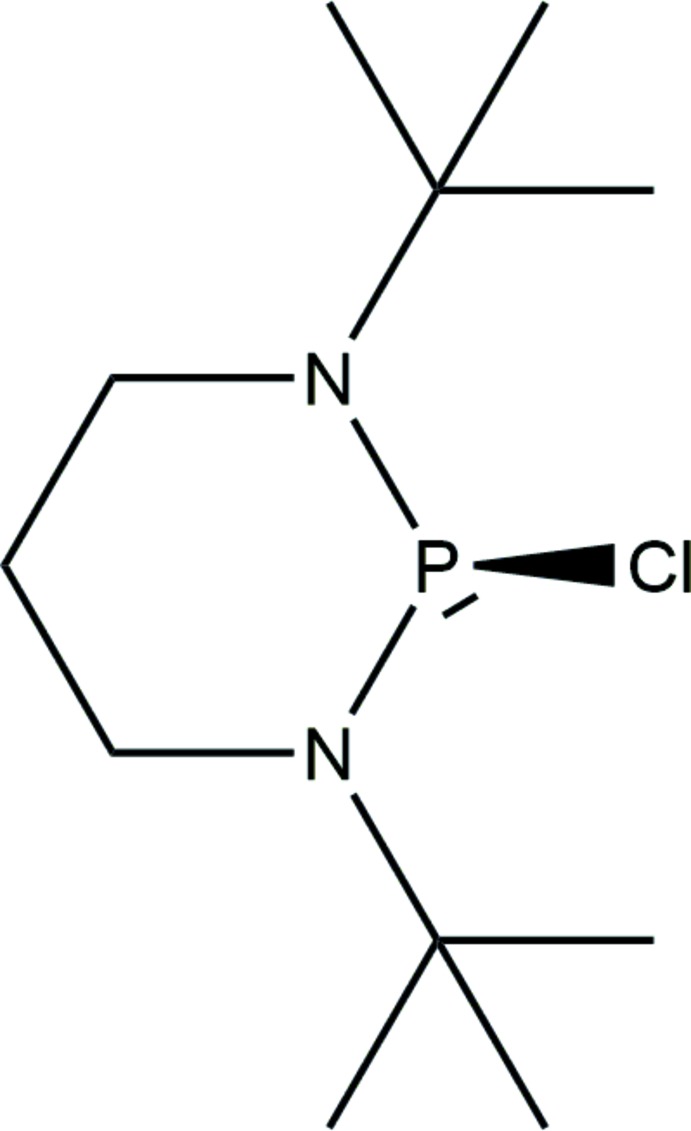


## Structural commentary   

**Figure 1:**
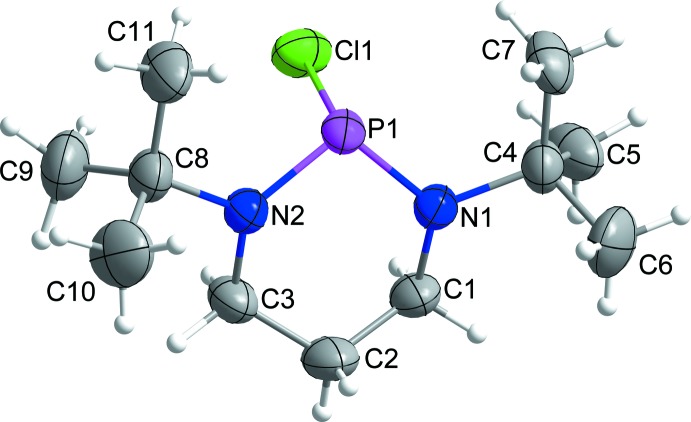
Diagram of the mol­ecular structure of compound **1** in the crystal displaying the atom-labelling scheme. Anisotropic displacement ellipsoids are drawn at the 50% probability level, the radii of hydrogen atoms are chosen arbitrarily.

**Figure 2:**
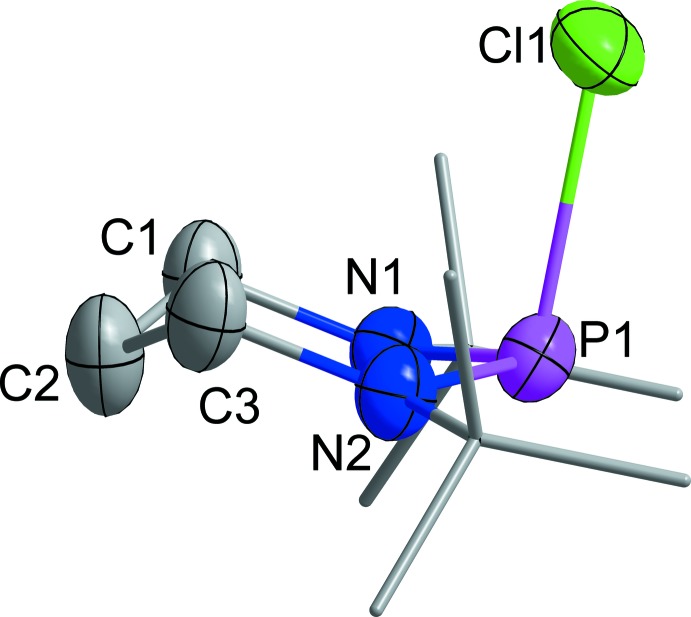
Chair conformation of the mol­ecule (H atoms are omitted for clarity); note the cyclo­hexane-like conformation at the ‘carbon-atom side’ [folding angle 53.07 (15)° as compared to 54.5 (6)° in the ordered, monoclinic phase of C_6_H_12_ (Kahn *et al.*, 1973 ▸)] and the ‘semi-flattened’ conformation [folding angle 27.96 (7)°] at the ‘phospho­rus/nitro­gen-atom side’.

The mol­ecular structure of **1** in the crystal is shown in Fig. 1 ▸. The mol­ecule does not suffer from conformational disorder, which is often recognized in the solids of saturated *N*-heterocyclic compounds. The main characteristics of the mol­ecule are: (i) the partially flattened chair conformation of the central six-membered heterocycle (displayed in more detail in Fig. 2 ▸) with an angle of 53.07 (15)° between the plane defined by the carbon atoms and the best plane of C1, C3, N1 and N2, and an angle of 27.96 (7)° between the latter plane and the plane defined by the nitro­gen and phospho­rus atoms; (ii) the equatorial orientation of both *tert*-butyl groups, enforced by the approximate trigonal–planar coordination of the nitro­gen atoms [sums of angles 356.2 (N1) and 355.8 (N2)], in combination with the axial orientation of the chloro substituent (Fig. 2 ▸) [out of plane angle: 106.83 (5)°]; (iii) the length of the P1—Cl1 bond, 2.2869 (6) Å, is substanti­ally longer than the standard single bond (2.02 Å; Brown, 2016 ▸) and the longest bond found in a six-membered NHPCl so far. The P—N bond lengths [P1—N1 = 1.6584 (14) and P1—N2 = 1.6519 (14) Å] are significantly smaller than the standard single-bond length [P—N = 1.704 (4) Å; Brown & Altermatt, 1985 ▸] and are close to the lower limit of the range found for NHPCls. The P—Cl bond is substanti­ally longer than the P—Cl single-bond length in PCl_3_ (2.034 Å; Galy & Enjalbert, 1982 ▸). The closest related five-membered NHPCl, 2-chloro-1,3-di-*tert*-butyl-2,1,3-phospha­diazo­lidine (CH_2_N^*t*^Bu)_2_>P–Cl shows almost identical bonding at the phospho­rus atom [P—N = 1.652 (2) and P—Cl = 2.3136 (7) Å; Denk *et al.*, 1999 ▸]. Unfortunately, a similar close relationship cannot be found among the known crystal structures of four-membered NHPCls and the closest related compound seems to be the *P*-chloro-substituted di­aza­phosphasiletidine Cl—P<(N^*t*^Bu)_2_>SiMe_2_ [P—N = 1.6815 (14) and P—Cl = 2.2498 (6) Å; Gün *et al.*, 2017 ▸].

A more general comparison with other *P*-chloro-functionalized six-membered heterocyclic phospho­rus compounds illustrates the P—Cl bond-length variation depending on the bonding situation in the heterocycle. Di-(3-methyl­indol-2-yl)chloro­phosphine-4-bromo­phenyl­methane (Mallov *et al.*, 2012 ▸), exhibits a planar coordination at the two carbon atoms next to the nitro­gen atoms due to exo­alkyl­ene group bonding, with a P—Cl bond length of only 2.108 (2) Å. In 2-chloro-1,3,5,7-tetra­methyl-4,6,8-trioxa-2-phosphaadamantane (Downing *et al.*, 2008 ▸), which can be considered as a chloro­phospho­rinane [(–C*R*)_2_>P—Cl] with an enforced chair conformation, P—Cl = 2.0754 (11) Å and in the 2-chloro-1,3,2-dioxaphophorinane derivative [(–O)_2_ >P—Cl] described by Pavan Kumar & Kumara Swamy (2007 ▸), P—Cl = 2.1227 (9) Å. Some examples of six-membered heterocycles with enforced ring flattening as a result of sterically demanding substituents (Brazeau *et al.*, 2012 ▸; Burford *et al.*, 2004 ▸; Holthausen *et al.*, 2016 ▸; Schranz *et al.*, 2000 ▸) and with flattening due to π-system involvement of the carbon atoms, such as 2-chloro-1,2,3,4-tetra­hydro-1,3,2-di­aza­phosphinium salts (Lesikar *et al.*, 2007 ▸; Vidovic *et al.*, 2006 ▸), 2-chloro-5,6-benzo-1,3,2-di­aza­phospho­rin-4-one (Sonnenburg *et al.*, 1997 ▸) and 2-chloro-2,3-di­hydro-1*H*-naphtho­[1,8-*de*][1,3,2]di­aza­phosphinines (Kozma *et al.*, 2015 ▸; Spinney *et al.*, 2007 ▸) all show significantly shorter P—Cl bonds compared to **1**, ranging from 2.072 (4) to 2.244 (3) Å. Further geometric details of **1** are given in the supporting information. C—C and C—N bond lengths, as well as endocyclic and exocyclic bond angles, are as expected taking into account the main structural characteristics given above. Finally it should be noted that the crystal structure determin­ation described here confirms the suggestions of Hutchins *et al.* (1972 ▸) concerning the structure of 2-chloro-1,3,2-di­aza­phophorinanes, derived by NMR spectroscopy.

## Supra­molecular features   

**Figure 3:**
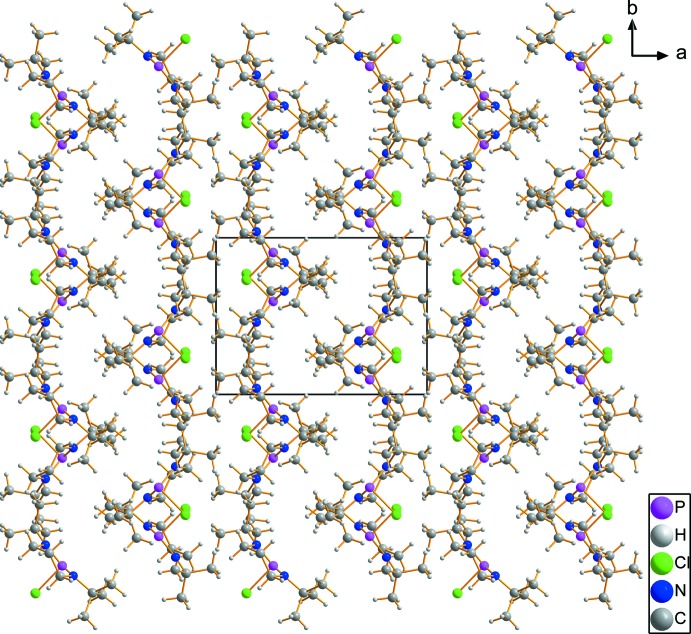
Packing diagram of **1** (view direction [00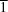]) showing a wave-like pattern. Inspection of the inter­molecular distances gives no evidence for inter­actions stronger than van der Waals forces and inter­molecular influence on the P—Cl bonding can be excluded.

Inspection of the inter­molecular distances gives no evidence for inter­actions stronger than van der Waals forces in the crystal of **1**. The closest contact is given between Cl1 and the methyl­ene group of the neighbouring mol­ecule containing C1 at a Cl⋯C distance of 3.7134 (18) Å, symmetry related by the *c* glide plane (symmetry code: *x*, 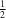 − *y*, 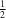 + *z*). Fig. 3 ▸ shows the packing of the mol­ecules in the crystal. Space group-symmetry gives rise to an appealing wave-like pattern.

## Database survey   

A search of the Cambridge Structural Database (Version 5.40, November 2018 update; Groom *et al.*, 2016 ▸) for the heterocycle substructure of 2-chloro-1,3,2-di­aza­phospho­rinanes (*i.e.* exclusively single bonds in the six-membered ring) yielded only one structure (DEHZOH; Mallov *et al.*, 2012 ▸). However, two of the ring carbon atoms are bonded to exo­alkyl­ene groups and are in planar coordination. A more general search allowing for alternative *P* ^III^-functionalization gave eight hits including *N* ^1^,*N* ^11^:*N* ^4^,*N* ^8^-bis­(μ_2_-methyl­phosphino)-1,4,8,11-tetra­aza­cyclo­tetra­decane (COLZUY; Hope *et al.*, 1984 ▸), 1,3-di-*t*ert-butyl-2-tri­phenyl­silyl-1,3,2-di­aza­phospho­rinane (DOD­DUV; Nifant’ev *et al.*, 1985 ▸), the 1,3-di-*tert*-butyl-1,3,2-di­aza­phospho­rinan­yloxy)calix(4)arenes FEMLOZ and FEMLUF (Maslennikova *et al.*, 2004 ▸), (η^5^-cyclo­penta­dien­yl)di­chloro­(1,3-dimethyl-1,3,2-di­aza­phosphol­yl)titanium (LAR­TED; Nifant’ev *et al.*, 1991 ▸), the phosphatris(pyrrol­yl)- and -(indol­yl)methanes NEQBUG (Barnard & Mason, 2001*a* ▸) and YETDIK (Barnard & Mason, 2001*b* ▸) and finally 3-(*tert*-but­yl)tri­methyl­silyl­amino-2,4-di-*tert*-butyl-1-[2-(1,3-di-*tert*-butyl-1,3,2-di­aza­phospho­ridin­yl)]imino-3-thio-1,2,4,3-thiadi­aza­phos­phetidine (YOVYEN; Wrackmeyer *et al.*, 1994 ▸). A search for *P*-chloro-functionalized six-membered ring compounds with any other three ring atoms joining the Cl—P<(N*R*)_2_ fragment and allowing for any kind of bonding in the ring gave 16 hits including eight with three carbon atoms. In addition to DEHZOH mentioned before, these include 2-chloro-1-(2′-chloro­eth­yl)-3-methyl-5,6-benzo-1,3,2-di­aza­phospho­rin-4-one (MAMBUX; Sonnenburg *et al.*, 1997 ▸), the 2-chloro-1,3-diorganyl-2,3-di­hydro-1*H*-naphtho­[1,8-*de*][1,3,2]di­aza­phos­phinines OGOXAL (Kozma *et al.*, 2015 ▸), REQKEE and TIPVIY (Spinney *et al.*, 2007 ▸) and the 1,3-bis­(2,6-di-iso­propyl­phen­yl)-2-chloro-1,2,3,4-tetra­hydro-1,3,2-di­aza­phosphinium salts NIJXUA (Lesikar *et al.*, 2007 ▸) and PENNUS (Vidovic *et al.*, 2006 ▸).

## Synthesis and crystallization   

The title compound was prepared under an argon atmosphere in oven-dried glassware using standard Schlenk techniques, modifying a published procedure (Nifant’ev *et al.*, 1977 ▸) by including a li­thia­tion step. 3.75 g (20.1 mmol) of *N*,*N′*-di-*tert*-butyl-1,3-propanedi­amine were dissolved in a mixture of diethyl ether and *n*-hexane (35 ml/55 ml). 16 ml of an *n*-butyl­lithium solution (*c* = 2.5 mol l^−1^ in *n*-hexane, 40 mmol) were slowly added at 263 K. Half an hour later, the reaction mixture was allowed to reach room temperature and the resulting pale-yellow suspension was stirred for 16 h. 2.92 g of PCl_3_ (21.3 mmol) were added dropwise over a period of 15 minutes at 195 K. To complete the reaction, the yellow reaction mixture was stirred for another hour with cooling and finally for two h at room temperature. Subsequently, the LiCl precipitate was filtered off and, after removal of the solvent under reduced pressure, the crude product was obtained as a yellow solid. Colourless block-shaped crystals suitable for X-ray structure determination were obtained by sublimation in a vacuum (3·10^−2^ mbar) at 313 K (30% yield; m.p. 327 K), by NMR analysis proved to be pure substance. ^1^H-NMR (300 MHz, CDCl_3_, 298 K) *δ* 3.16–3.07 (*m*, 4 H), 1.90–1.80 (*m*, 2 H), 1.34 [*d*, ^4^ *J*(H,P) = 3.5 Hz, 18H].

## Refinement   

Crystal data, data collection and structure refinement details are summarized in Table 1 ▸. Positions of all hydrogen atoms were identified *via* subsequent Δ*F* syntheses. In the refinement, a riding model was applied using idealized C—H bond lengths (0.98–0.99 Å) as well as H—C—H and C—C—H angles. In addition, the H atoms of the CH_3_ groups were allowed to rotate around the neighbouring C—C bonds. The *U*_iso_ values were set to 1.5*U*_eq_(C_meth­yl_) and 1.2*U*_eq_(C_methyl­ene_).

## Supplementary Material

Crystal structure: contains datablock(s) I. DOI: 10.1107/S2056989019004195/pk2615sup1.cif

Structure factors: contains datablock(s) I. DOI: 10.1107/S2056989019004195/pk2615Isup2.hkl

Supporting information file. DOI: 10.1107/S2056989019004195/pk2615Isup3.cml

CCDC reference: 1906304

Additional supporting information:  crystallographic information; 3D view; checkCIF report

## Figures and Tables

**Table 1:** Experimental details

Crystal data	
Chemical formula	C_11_H_24_ClN_2_P
*M*_r_	250.74
Crystal system, space group	Monoclinic, *P*2_1_/*c*
Temperature (K)	173
*a*, *b*, *c* (Å)	12.5954 (5), 9.1549 (3), 12.9614 (6)
β (°)	101.547 (3)
*V* (Å^3^)	1464.33 (10)
*Z*	4
Radiation type	Mo *K*α
μ (mm^−1^)	0.35
Crystal size (mm)	0.48 × 0.28 × 0.25

Data collection	
Diffractometer	Stoe IPDS II
Absorption correction	Multi-scan (*XPREP*; Bruker, 2008 ▸)
*T*_min_, *T*_max_	0.761, 0.929
No. of measured, independent and observed [*I* > 2σ(*I*)] reflections	16291, 3943, 3547
*R*_int_	0.050
(sin θ/λ)_max_ (Å^−1^)	0.686

Refinement	
*R*[*F* ^2^ > 2σ(*F* ^2^)], *wR*(*F* ^2^), *S*	0.048, 0.109, 1.01
No. of reflections	3943
No. of parameters	142
H-atom treatment	H-atom parameters constrained
Δρ_max_, Δρ_min_ (e Å^−3^)	0.41, −0.21

Computer programs: *X-AREA* (Stoe & Cie, 2002 ▸), *SHELXT* (Sheldrick, 2015*a* ▸), *SHELXL2014* (Sheldrick, 2015*b* ▸) and *DIAMOND* (Brandenburg, 2015 ▸).

## supplementary crystallographic information

### Crystal data

**Table:**

C_11_H_24_ClN_2_P	*F*(000) = 544
*M**_r_* = 250.74	*D*_x_ = 1.137 Mg m^−^^3^
Monoclinic, *P*2_1_/*c*	Mo *K*α radiation, λ = 0.71073 Å
*a* = 12.5954 (5) Å	Cell parameters from 20870 reflections
*b* = 9.1549 (3) Å	θ = 4.5–59.2°
*c* = 12.9614 (6) Å	µ = 0.35 mm^−^^1^
β = 101.547 (3)°	*T* = 173 K
*V* = 1464.33 (10) Å^3^	Block, colourless
*Z* = 4	0.48 × 0.28 × 0.25 mm

### Data collection

**Table:**

Stoe IPDS II diffractometer	3547 reflections with *I* > 2σ(*I*)
ω–scans	*R*_int_ = 0.050
Absorption correction: multi-scan (XPREP; Bruker, 2008)	θ_max_ = 29.2°, θ_min_ = 2.7°
*T*_min_ = 0.761, *T*_max_ = 0.929	*h* = −17→17
16291 measured reflections	*k* = −12→12
3943 independent reflections	*l* = −17→17

### Refinement

**Table:**

Refinement on *F*^2^	Primary atom site location: structure-invariant direct methods
Least-squares matrix: full	Secondary atom site location: difference Fourier map
*R*[*F*^2^ > 2σ(*F*^2^)] = 0.048	Hydrogen site location: difference Fourier map
*wR*(*F*^2^) = 0.109	H-atom parameters constrained
*S* = 1.01	*w* = 1/[σ^2^(*F*_o_^2^) + (0.0327*P*)^2^ + 0.8644*P*] where *P* = (*F*_o_^2^ + 2*F*_c_^2^)/3
3943 reflections	(Δ/σ)_max_ = 0.001
142 parameters	Δρ_max_ = 0.41 e Å^−^^3^
0 restraints	Δρ_min_ = −0.21 e Å^−^^3^

### Special details

**Table:**

Geometry. All esds (except the esd in the dihedral angle between two l.s. planes) are estimated using the full covariance matrix. The cell esds are taken into account individually in the estimation of esds in distances, angles and torsion angles; correlations between esds in cell parameters are only used when they are defined by crystal symmetry. An approximate (isotropic) treatment of cell esds is used for estimating esds involving l.s. planes.

### Fractional atomic coordinates and isotropic or equivalent isotropic displacement parameters (Å^2^)

**Table:**

	*x*	*y*	*z*	*U*_iso_*/*U*_eq_	
Cl1	0.85529 (4)	0.26976 (5)	0.83702 (4)	0.05736 (15)	
P1	0.72874 (3)	0.09412 (5)	0.77742 (3)	0.03774 (11)	
N1	0.67636 (11)	0.15879 (16)	0.65862 (11)	0.0411 (3)	
N2	0.80820 (11)	−0.03915 (15)	0.75215 (11)	0.0390 (3)	
C1	0.73831 (16)	0.1599 (2)	0.57350 (14)	0.0511 (4)	
H11	0.7950	0.2362	0.5881	0.061*	
H12	0.6892	0.1842	0.5060	0.061*	
C2	0.79040 (18)	0.0142 (3)	0.56389 (15)	0.0579 (5)	
H21	0.7332	−0.0608	0.5451	0.070*	
H22	0.8314	0.0187	0.5063	0.070*	
C3	0.86546 (15)	−0.0296 (2)	0.66384 (14)	0.0485 (4)	
H31	0.8981	−0.1256	0.6538	0.058*	
H32	0.9248	0.0428	0.6809	0.058*	
C4	0.58516 (16)	0.2663 (2)	0.64533 (15)	0.0507 (4)	
C5	0.6238 (2)	0.4171 (3)	0.6188 (2)	0.0810 (7)	
H51	0.6437	0.4141	0.5495	0.122*	
H52	0.6870	0.4457	0.6722	0.122*	
H53	0.5654	0.4883	0.6176	0.122*	
C6	0.49516 (19)	0.2120 (3)	0.5566 (2)	0.0804 (8)	
H61	0.5245	0.1962	0.4930	0.121*	
H62	0.4372	0.2850	0.5423	0.121*	
H63	0.4660	0.1199	0.5777	0.121*	
C7	0.5396 (2)	0.2788 (3)	0.7459 (2)	0.0756 (7)	
H71	0.5958	0.3169	0.8030	0.113*	
H72	0.5165	0.1822	0.7653	0.113*	
H73	0.4774	0.3454	0.7337	0.113*	
C8	0.85809 (15)	−0.1413 (2)	0.83911 (15)	0.0489 (4)	
C9	0.97954 (16)	−0.1119 (3)	0.87176 (17)	0.0655 (6)	
H91	1.0154	−0.1390	0.8141	0.098*	
H92	1.0096	−0.1700	0.9343	0.098*	
H93	0.9915	−0.0079	0.8879	0.098*	
C10	0.8368 (3)	−0.2973 (2)	0.7984 (2)	0.0860 (8)	
H101	0.7585	−0.3146	0.7800	0.129*	
H102	0.8699	−0.3664	0.8533	0.129*	
H103	0.8684	−0.3110	0.7359	0.129*	
C11	0.80696 (18)	−0.1203 (3)	0.93605 (17)	0.0631 (6)	
H111	0.7286	−0.1367	0.9164	0.095*	
H112	0.8208	−0.0205	0.9629	0.095*	
H113	0.8388	−0.1902	0.9908	0.095*	

### Atomic displacement parameters (Å^2^)

**Table:**

	*U*^11^	*U*^22^	*U*^33^	*U*^12^	*U*^13^	*U*^23^
Cl1	0.0692 (3)	0.0498 (3)	0.0529 (3)	−0.0125 (2)	0.0117 (2)	−0.0122 (2)
P1	0.0374 (2)	0.0386 (2)	0.0394 (2)	0.00313 (16)	0.01304 (15)	0.00418 (16)
N1	0.0410 (7)	0.0420 (7)	0.0407 (7)	0.0073 (6)	0.0093 (5)	0.0033 (6)
N2	0.0388 (7)	0.0362 (6)	0.0423 (7)	0.0030 (5)	0.0093 (5)	0.0012 (5)
C1	0.0553 (10)	0.0609 (11)	0.0386 (8)	0.0111 (9)	0.0133 (7)	0.0070 (8)
C2	0.0666 (12)	0.0672 (13)	0.0426 (9)	0.0142 (10)	0.0169 (9)	−0.0053 (9)
C3	0.0493 (9)	0.0537 (10)	0.0451 (9)	0.0109 (8)	0.0158 (7)	−0.0028 (8)
C4	0.0511 (10)	0.0481 (10)	0.0529 (10)	0.0149 (8)	0.0106 (8)	0.0062 (8)
C5	0.101 (2)	0.0493 (12)	0.0943 (18)	0.0181 (13)	0.0239 (15)	0.0167 (12)
C6	0.0526 (12)	0.098 (2)	0.0830 (16)	0.0202 (13)	−0.0058 (11)	−0.0045 (15)
C7	0.0733 (15)	0.0876 (17)	0.0719 (14)	0.0409 (14)	0.0291 (12)	0.0144 (13)
C8	0.0512 (10)	0.0434 (9)	0.0522 (10)	0.0103 (8)	0.0110 (8)	0.0108 (8)
C9	0.0477 (10)	0.0957 (17)	0.0521 (11)	0.0230 (11)	0.0076 (8)	0.0079 (11)
C10	0.115 (2)	0.0402 (11)	0.102 (2)	0.0101 (13)	0.0214 (17)	0.0085 (12)
C11	0.0636 (12)	0.0692 (13)	0.0612 (12)	0.0141 (10)	0.0234 (10)	0.0278 (10)

### Geometric parameters (Å, º)

**Table:**

Cl1—P1	2.2869 (6)	C5—H53	0.9800
P1—N2	1.6519 (14)	C6—H61	0.9800
P1—N1	1.6584 (14)	C6—H62	0.9800
N1—C1	1.473 (2)	C6—H63	0.9800
N1—C4	1.496 (2)	C7—H71	0.9800
N2—C3	1.472 (2)	C7—H72	0.9800
N2—C8	1.502 (2)	C7—H73	0.9800
C1—C2	1.502 (3)	C8—C10	1.527 (3)
C1—H11	0.9900	C8—C9	1.527 (3)
C1—H12	0.9900	C8—C11	1.534 (3)
C2—C3	1.498 (3)	C9—H91	0.9800
C2—H21	0.9900	C9—H92	0.9800
C2—H22	0.9900	C9—H93	0.9800
C3—H31	0.9900	C10—H101	0.9800
C3—H32	0.9900	C10—H102	0.9800
C4—C5	1.526 (3)	C10—H103	0.9800
C4—C6	1.527 (3)	C11—H111	0.9800
C4—C7	1.529 (3)	C11—H112	0.9800
C5—H51	0.9800	C11—H113	0.9800
C5—H52	0.9800		
			
N2—P1—N1	102.93 (7)	H52—C5—H53	109.5
N2—P1—Cl1	100.22 (5)	C4—C6—H61	109.5
N1—P1—Cl1	100.57 (6)	C4—C6—H62	109.5
C1—N1—C4	114.76 (14)	H61—C6—H62	109.5
C1—N1—P1	121.73 (11)	C4—C6—H63	109.5
C4—N1—P1	119.69 (12)	H61—C6—H63	109.5
C3—N2—C8	115.07 (13)	H62—C6—H63	109.5
C3—N2—P1	121.39 (12)	C4—C7—H71	109.5
C8—N2—P1	119.34 (11)	C4—C7—H72	109.5
N1—C1—C2	111.20 (16)	H71—C7—H72	109.5
N1—C1—H11	109.4	C4—C7—H73	109.5
C2—C1—H11	109.4	H71—C7—H73	109.5
N1—C1—H12	109.4	H72—C7—H73	109.5
C2—C1—H12	109.4	N2—C8—C10	107.76 (17)
H11—C1—H12	108.0	N2—C8—C9	110.10 (16)
C3—C2—C1	112.13 (16)	C10—C8—C9	110.9 (2)
C3—C2—H21	109.2	N2—C8—C11	110.81 (15)
C1—C2—H21	109.2	C10—C8—C11	109.06 (19)
C3—C2—H22	109.2	C9—C8—C11	108.19 (17)
C1—C2—H22	109.2	C8—C9—H91	109.5
H21—C2—H22	107.9	C8—C9—H92	109.5
N2—C3—C2	111.45 (15)	H91—C9—H92	109.5
N2—C3—H31	109.3	C8—C9—H93	109.5
C2—C3—H31	109.3	H91—C9—H93	109.5
N2—C3—H32	109.3	H92—C9—H93	109.5
C2—C3—H32	109.3	C8—C10—H101	109.5
H31—C3—H32	108.0	C8—C10—H102	109.5
N1—C4—C5	110.44 (17)	H101—C10—H102	109.5
N1—C4—C6	108.04 (17)	C8—C10—H103	109.5
C5—C4—C6	110.3 (2)	H101—C10—H103	109.5
N1—C4—C7	111.19 (15)	H102—C10—H103	109.5
C5—C4—C7	108.4 (2)	C8—C11—H111	109.5
C6—C4—C7	108.5 (2)	C8—C11—H112	109.5
C4—C5—H51	109.5	H111—C11—H112	109.5
C4—C5—H52	109.5	C8—C11—H113	109.5
H51—C5—H52	109.5	H111—C11—H113	109.5
C4—C5—H53	109.5	H112—C11—H113	109.5
H51—C5—H53	109.5		
			
N2—P1—N1—C1	−33.19 (16)	C1—C2—C3—N2	59.3 (2)
Cl1—P1—N1—C1	69.98 (15)	C1—N1—C4—C5	−48.7 (2)
N2—P1—N1—C4	170.01 (13)	P1—N1—C4—C5	109.66 (18)
Cl1—P1—N1—C4	−86.82 (14)	C1—N1—C4—C6	72.0 (2)
N1—P1—N2—C3	33.52 (15)	P1—N1—C4—C6	−129.63 (17)
Cl1—P1—N2—C3	−69.94 (13)	C1—N1—C4—C7	−169.02 (19)
N1—P1—N2—C8	−170.64 (13)	P1—N1—C4—C7	−10.7 (2)
Cl1—P1—N2—C8	85.90 (13)	C3—N2—C8—C10	−72.3 (2)
C4—N1—C1—C2	−153.99 (17)	P1—N2—C8—C10	130.43 (17)
P1—N1—C1—C2	48.1 (2)	C3—N2—C8—C9	48.8 (2)
N1—C1—C2—C3	−58.7 (2)	P1—N2—C8—C9	−108.48 (16)
C8—N2—C3—C2	154.03 (17)	C3—N2—C8—C11	168.49 (17)
P1—N2—C3—C2	−49.2 (2)	P1—N2—C8—C11	11.2 (2)
